# Relationship between Spiritual Intelligence and Professional Self-concept among Iranian Nurses

**DOI:** 10.17533/udea.iee.v39n3e12

**Published:** 2021-11-08

**Authors:** Mohsen Hojat, Zohreh Badiyepeymaiejahromi

**Affiliations:** 1 . Nurse, Ph.D. Assistant Professor. Jahrom University of Medical Sciences, Jahrom, Iran Email: mohsenhojat@yahoo.com Iran University of Medical Sciences Jahrom University of Medical Sciences Jahrom Iran mohsenhojat@yahoo.com; 2 . Nurse, Ph.D. Assistant Professor. Jahrom University of Medical Sciences, Jahrom, Iran Email: z.badiyepeyma@gmail.com. Corresponding author. Iran University of Medical Sciences Jahrom University of Medical Sciences Jahrom Iran z.badiyepeyma@gmail.com

**Keywords:** self concept, spiritualism, intelligence, nurses, Iran., autoimagen, espiritualismo, inteligencia, enfermeras y enfermeros, Iran., autoimagem, spiritualismo, inteligência, enfermeiras y enfermeiros, Iran.

## Abstract

**Objective:**

To determine the relationship between spiritual intelligence (SI) and professional self-concept (PSC) among Iranian nurses.

**Methods:**

This is a correlation study. A convenience sampling method was used and 344 nurses were selected from hospitals of Jahrom University of Medical Sciences. Data collection standard tools included two validated scales: Cowin’s Nurse Self-Concept Questionnaire (36 items scored ranged from 1 to 8; Maximum score=288; 6 subcategories: General Nurse Self-Concept, Knowledge, Care, Communication, Staff Relation and Leadership) and Abdollahzadeh’s SI Questionnaire (29 items scored ranged from 0 to 5; Maximum score=145; 2 subcategories: Relying on the inner core and Understanding and communicating with the origin of the universe**.**

**Results:**

The mean total score of PSC was 220.3±30.61 and 120.67±16.13 for SI. There was a significant statistical correlation between PSC (r=0.348, *p*<0.0001) and almost all subcategories and SI. The results of the regression analysis showed that SI predicts 13.3% of the variance of PSC (*p*<0.0001).

**Conclusion:**

Considering the correlation of SI and PSC among Iranian nurses, it is suggested that strategies be used to train and promote the SI of nurses.

## Resumen

**Objetivo:**

Determinar la relación entre la inteligencia espiritual y el autoconcepto profesional entre las enfermeras iraníes.

**Métodos:**

Se trata de un estudio correlacional. Se utilizó un método de muestreo por conveniencia y se seleccionaron 344 enfermeras de los hospitales de la Universidad de Ciencias Médicas de Jahrom. Los instrumentos de recolección de los datos incluían dos escalas validadas: Cuestionario de autoconcepto de la enfermera de Cowin (36 ítems con puntuaciones que van de 1 a 8; puntuación máxima=288; 6 subcategorías: Autoconcepto enfermero general, Conocimiento, Cuidados, Comunicación, Relación con el personal y Liderazgo) y el Cuestionario de Inteligencia Espiritual de Abdollahzadeh (29 ítems puntuados de 0 a 5; Puntuación máxima=145; 2 subcategorías: Apoyarse en el núcleo interno y Comprender y comunicarse con el origen del universo).

**Resultados:**

La puntuación total media del Autoconcepto Profesional fue de 220.3±30.61 y de 120.67±16.13 para Inteligencia Espiritual. Hubo una correlación estadística significativa entre el puntaje total de la escala Autoconcepto Professional (r=0.348, *p*<0.0001) y de casi todas sus subcategorías, con la Inteligencia Espiritual. Los resultados del análisis de regresión mostraron que la Inteligencia Espiritual predice el 13.3% de la varianza del autoconcepto profesional (*p*<0.0001).

**Conclusión:**

Existe correlación entre la Inteligencia Espiritual y el Autoconcepto Profesional entre las enfermeras iraníes. En este sentido, se sugiere que se utilicen estrategias para formar y promover la Inteligencia Espiritual de las enfermeras.

**Objetivo:**

Determinar a relação entre inteligência espiritual e autoconceito profissional entre enfermeiras iranianas.

**Métodos:**

Este é um estudo correlacional. Um método de amostragem de conveniência foi usado e 344 enfermeiras foram selecionadas dos hospitais da Jahrom University of Medical Sciences. Os instrumentos de coleta de dados incluíram duas escalas validadas: Questionário de autoconceito do enfermeiro de Cowin (36 itens com pontuação variando de 1 a 8; pontuação máxima = 288; 6 subcategorias: Autoconceito geral de enfermagem, Conhecimento, Cuidado, Comunicação, Relacionamento com a equipe e Liderança) e o Questionário de Inteligência Espiritual de Abdollahzadeh (29 itens pontuados de 0 a 5; Pontuação máxima = 145; 2 subcategorias: Baseando-se no núcleo interno e entendendo e se comunicando com a origem do universe.

**Resultados:**

A pontuação total média para o profissional do autoconceito foi de 220.3 ± 30.61 e 120.67 ± 16.13 para Inteligência Espiritual, houve correlação estatisticamente significante entre a pontuação total da escala de Autoconceito Profissional (r = 0.348, *p* <0.0001) e quase todas as suas subcategorias, com Inteligência Espiritual. Os resultados da análise de regressão mostraram que a Inteligência Espiritual prediz 13.3% da variância do autoconceito ou profissional (*p* <0.0001).

**Conclusão:**

Existe uma correlação entre Inteligência Espiritual e Autoconceito Profissional entre os enfermeiros iranianos,

## Introduction

The importance of professional self-concept (PSC) in nursing studies has been recognized.(1) The nursing PSC is defined as the nurse's emotions to her/himself which is influenced by the characteristics, regulations, and values of the nursing discipline. It leads to individual thinking, acting, and feeling like a nurse.(2) Ignoring the importance of PSC can be harmful to the nursing profession because positive PSC plays an important role in shaping the professional identity.(3) It can increase the efficiency of the individual profession, increase job satisfaction, and predict the newly graduated nurses stay in the nursing profession.(4) But when PSC is not developed, job burnout(5) and workplace bullying will increase,(6) and patient safety will decrease.(7) The results of a study revealed that various factors such as nursing education, nursing image, professional values, and sociocultural environment can affect PSC.(7) Since PSC is a complex, and context-based phenomenon, all related variables need to be studied.(8)

Spiritual intelligence (SI) is one of the controversial and novel topics that has been developed because of the interest and attention of researchers.(9) SI does not consider a specific religious propensity. It is a type of ability that causes self-control, self-consciousness, increased peace, purposefulness, profound understanding of life meaning, and constructive communication with others.(10) SI has been explained as the potency to do with cognition and humanity while retaining internal and external tranquility, disregard the situation.(11)

Awareness and understanding of SI can be an important strategy for promoting human resources in the workplace.(12) SI helps nurses to give meaningful services, and cope better with work pressures.(13) It also allows nurses to achieve fundamental life goals, and produce individual meanings. Nurses with a higher SI not only can respond appropriately in specific situations, but also they can understand why they are in that situation, and how to adapt it.(14) Similar to PSC, SI correlates with job satisfaction,(15) and nursing care behavior.(16) According to the introduction nurses' SI and PSC are among the important factors that can affect the way patients are cared for.^7^^,^^16^ All Iranian nurses in this study are Muslim. SI and PSC are related to the socio-cultural, and religious conditions of countries. Because individuals are influenced by their context interpretation of personal and professional life experiences.

A review of previous research shows that few studies on these two variables have been done separately, but no research has been found on the subject of PSC and SI. Considering that SI is one of the important issues that can be affected PSC, so this study aims to determine the relationship between PSC and SI among Iranian nurses. Emphasizing the inner characteristics of nurses and examining the SI of them can provide valuable information about their PSC.

## Methods

Study Design. This is a descriptive-correlational study, which was conducted in 2019 in teaching hospitals (Motahari, and Pymanie) affiliated with Jahrom University of Medical Sciences in Iran.

Participants. 344 nurses from general and intensive wards participated in the study by convenient sampling method. The inclusion criteria were at least six months of work experience, bachelor's degree, and staff nurse. Exclusion criteria were incomplete completion of the questionnaire.

Data gathering. The second author referred to the general and intensive wards of Motahari and Peymaneh hospitals in different shifts (the morning, evening, and night) between August and September 2019. The author invited nurses to participate in the study and obtained their consent. Then the author distributed the questionnaires among the nurses to complete in their free time. The completed questionnaires were collect by the author.

Variables. The following questionnaires were used to collect research data: (i) Nurse Self-concept Questionnaire (NSCQ): Cowin’s 36 items questionnaire includes six subscales such as general nurse self-concept, knowledge, care, staff relation, communication, and leadership. Each item was positively grad based on the 1-8 Likert scale. The range of scores is 36-288. Higher scores show better PSC (17). In the study of Zencir *et al.*(18) in Turkey, content validity, construct validity, convergent validity and discriminant validity was confirmed. Cronbach's alpha of this questionnaire was reported in different dimensions between 0.83-0.91. The validity and reliability of the Persian version of this questionnaire have also been confirmed. In the study of Badiyepeyma *et al.,*(19) the correlation coefficients of Spearman-Brown and Cronbach's alpha were 0.84 and 0.97, respectively. All subscales had a moderate or strong correlation and significant relationship with each other, which indicates the construct validity of this questionnaire; (ii) SI Questionnaire: This questionnaire was designed in Iran by Abdollahzadeh *et al.*(20) with 29 items. It has two subscales contains "understanding and communicating with the origin of the universe" with 12 questions, and "spiritual life or relying on the inner core" with 17 questions. Each item has been graded on a Likert scale from 1 to 5 (strongly disagree, disagree, somewhat, agree, and strongly agree). Scores range were 29 to 145. This questionnaire has been developed according to the cultural characteristics of Iranian society. Its validity and reliability have been confirmed. To evaluate the validity, in addition to the content and face validity of the questions, which were confirmed by experts, factor analysis was used and the correlation of all questions was above 0.3. The reliability of this questionnaire was 0.89.

*Statistical methods.* The data were analyzed based on descriptive (frequency, percentile, mean and standard deviation), and analytical statistics by SPSS.v.21 statistical software. The significance level was appointed 0.05. The normality of variables was evaluated by the Kolmogorov Smirnov test (*p*>0.05). Pearson Correlation test was used to examine the relationship between SI and PSC. Multivariate regression analysis (Enter model) was used to predict students' PSC based on the SI.

*Ethical Considerations.* This article is the result of a research project approved by the Ethics Committee at Jahrom University of Medical Sciences (IR.JUMS.REC.1393.142). The Helsinki Statement was followed in the study. Before starting the research, participants were informed about the goals of the research. Written consent was signed. The questionnaires were anonymously, and all data would be kept confidential.

## Results

344 nurses participated in the study. The mean and standard deviation of age and work experience were 28.44±6.58, 5.63±5.78 respectively. Most nurses were female (71.5%). Some socio-demographic statistics values are presented in Table 1. The mean of PSC was 220.3±30.61, and the mean of SI was 120.67±16.13 (Table 2).

**Table 1:** Socio-demographic characteristic of 344 nurses

Variable	Frequency	Percent
Gender		
Male	98	28.5
Female	246	71.5
Level of Education		
Bachelor of Science	327	95.1
Master of Science	17	4.9
Job Position		
Head nurses	36	10.5
Staff	308	89.5
Ward		
General	240	69.77
Intensive	104	30.23

**Table 2:** descriptive analysis of PSC and SI score

Scale / subcategories	Mean	Std. Deviation	Std. Error of Mean	Variance	Min	Max
Professional Self-concept (PSC)	220.30	30.61	1.65	937.39	87	287
General Nurse Self-Concept	37.20	8.02	0.43	64.45	6	48
Knowledge	29.22	7.59	0.40	57.73	12	48
Care	39.29	5.53	0.29	30.68	23	48
Communication	40.64	5.86	0.31	34.42	11	48
Staff Relation	39.38	6.97	0.37	48.68	6	48
Leadership	34.47	10.69	0.57	114.37	6	96
Spiritual Intelligence (SI)	120.66	16.13	0.872	260.23	60	145
Relying on the inner core	68.933	9.73	0.524	94.79	41	85
Understanding and communicating with the origin of the universe	51.728	7.41	0.401	54.94	17	60

There was a significant statistical correlation between PSC and SI (Pearson Correlation=0.34, Sig 2-tailed <0.0001) and for all subcategories of PSC and SI except leadership subcategory (Table 3).

**Table 3:** Relationship between subcategories of PSC and SI

PSC/SI	Understanding and communicating with the origin of the universe	Relying on the inner core
General Nurse Self-Concept	r=0.361	r=0.309
General Nurse Self-Concept	*p*<0.001	*p*<0.001
Knowledge	r=0.227	r=0.183
Knowledge	*p*<0.001	*p*=0.001
Care	r=0.178	r=0.117
Care	*p*<0.001	*p*=0.031
Communication	r=0.338	r=0.263
Communication	*p*<0.001	*p*<0.001
Staff Relation	r=0.0399	r=0.293
Staff Relation	*p*<0.001	*p*<0.001
Leadership	r=0.076	r=0.038
Leadership	*p*=0.161	*p*=0.485

One-way variance ANOVA analysis reveals that variables are eligible for multivariate linear regression testing (*df*=2, *F*=25.996, *p-*value<0.0001). According to the results of Table 4, the significance level of understanding and communicating with the origin of the universe is higher than the significance level (0.05). Consequently, in this dimension, the understanding and communicating with the origin of the universe is estimated to be the same, and the assumption of the test based on the difference in variables not accepted in a 95% confidence interval. However, the significance level of relying on the inner core is less than (0.05). Predictor variables can predict 13.3% of PSC variance.

**Table 4:** Multivariate regression analysis of the Enter model for predicting PSC through subcategories S**I**

Variables	B	SE	Beta	t	***p*-value**
Constant	141.67	11.71		12.09	<0.0001
Relying on the inner core	1.15	0.24	0.36	4.72	<0.0001
Understanding and communicating with the origin of the universe	-0.011	0.32	-0.003	-0.03	0.972
R=0.365a R2 = 0.133 Adjusted R2 =0.128	R=0.365a R2 = 0.133 Adjusted R2 =0.128	R=0.365a R2 = 0.133 Adjusted R2 =0.128	R=0.365a R2 = 0.133 Adjusted R2 =0.128	R=0.365a R2 = 0.133 Adjusted R2 =0.128	R=0.365a R2 = 0.133 Adjusted R2 =0.128

^a. Predictors: (Constant), understanding and communicating with the origin of the universe, relying on the inner core^

**Figure 1:**
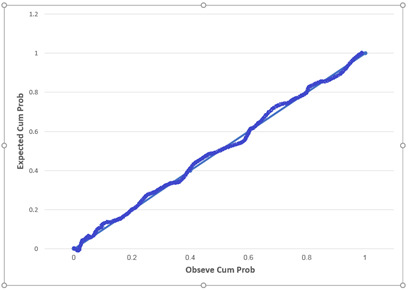
Normal P-P plot of regression standardized residual

Figure 1 shows a positive linear relationship and the residuals are relatively normally distributed.

## Discussion

The results of this study showed that SI is related to nurses' PSC and SI explains 13.3% of the changes in PSC. Since no similar study was found to compare the correlation of PSC based on SI, the results of the present study were compared with related studies of SI and PSC. The results of Mosayebi *et al.*(21) showed that there is a negative linear correlation between job stress and nurses' PSC. As a result, with the reduction of job stress in nurses, their PSC increases. SI training is an effective way to reduce stress(22) and increase job satisfaction of nurses,(23) so using SI training has been suggested to reduce job stress and increase job satisfaction. According to the results of the above studies, it can be concluded that SI plays a role in increasing the PSC of nurses by reducing job stress and increasing job satisfaction. 

In this regard, the result of Ebrahimi Barmi *et al.*(9) showed that there is a significant relationship between spiritual intelligence and resiliency. According to Sahebalzamani*et al.,*(24) there is a significant relationship between SI with nurses' psychological well-being and having a purpose in life. So SI aids nurses to meliorate their psychological well-being and have a goal in life that may guide to the health procurement of them and patients.

The study of Rani *et al.*(25) also showed that there was a positive correlation between SI and nurses' job performance. The nurses with higher SI had better performance in their work. Based on the descriptions of SI, it can be concluded that people with high SI have more ability and flexibility, which is effective in improving job performance. SI helps people to do difficult things and makes their job difficulties an opportunity to help other people and be altruistic. On the other hand, SI by emphasizing positive inner and constructive motivations can also strengthen the PSC of nurses. Therefore, high SI both improves the job performance of nurses and, in turn, can improve the PSC. Also, the results of the studies have shown that SI is one of the factors affecting nurses' caring behaviors. In this regard, the study of Kaur *et al.* in Malaysia(16) also showed that promoting SI and strengthening nurses' beliefs can help improve the quality of patient care. The SI helps people to have a better understanding of goals and the right ways to achieve goals and to choose the right motivational orientations in life.

Findings of another study also showed a significant relationship between interest in the nursing profession and SI. Thus, the people who enter the nursing discipline with interest have a higher SI. It seems that SI can increase the problem-solving ability and flexibility against problems also dealing with stressful situations by nurses, and the reason for this is the better adaption to the conditions and work environment of nursing among interested people. It seems that people with higher SI are more likely to use adaptive problem-solving skills and use spiritual resources to solve problems in their daily lives and give meaning and value to their daily affairs. These people also use behaviors such as forgiveness, self-sacrifice, self-control, and sanctifying daily affairs more, and in this way, they can solve problems better, so they will have a high PSC.

Another study also showed the relationship between SI and happiness in nurses. The activities that nurses do in search of spirituality, such as helping others and caring for them can lead to happiness, and the belief that there are prominent forces and destinations in the world that can increase people's happiness and thus can affect their PSC positively. Nurses have their own beliefs and the mental image of their situation that they reflect in some way in their minds. This mental image is created by personal experiences and the impact of the professional world on the individual. According to this, they evaluate their life and profession and try to deal with it, so SI can affect PSC by influencing the feeling of happiness.

The present study had some limitations. This study was conducted only in educational hospitals of Jahrom University of Medical Sciences. The questionnaires were used to collect data. Although the present study is correlational and predictive, it suggested that other related variables can predict most changes in PSC examined. Also, pay more attention to experimental studies in this regard. SI is a predictive factor in the PSC of nurses. But this prediction was not high, so other factors for increasing PSC must be considered. According to the findings, SI is related to PSC and its dimensions. People with high SI have a holistic view of life and a greater ability to solve problems by enjoying positive moral virtues. Because nurses face many problems and stresses on a workday, SI can improve PSC and the quality of care. Therefore, the results of this study show the need to improve the level of SI of clinical nurses. Improving the SI of nurses during their education and presenting continuing education programs in this regard is recommended. This result could help professional staff, like physicians, pastoral/ spiritual care providers, social workers, and psychologists, to promote SI and PSC.
